# Effect of rehabilitation worker input on visual function outcomes in individuals with low vision: study protocol for a randomised controlled trial

**DOI:** 10.1186/s13063-016-1235-2

**Published:** 2016-02-24

**Authors:** Jennifer H. Acton, Bablin Molik, Alison Binns, Helen Court, Tom H. Margrain

**Affiliations:** School of Optometry & Vision Sciences, College of Biomedical and Life Sciences, Cardiff University, Maindy Road, Cardiff, CF24 4HQ Wales UK; Division of Optometry, School of Health Sciences, City University London, Northampton Square, London, EC1V 0HB England UK; Institute of Health and Wellbeing, University of Glasgow, 1st floor, Administration Building, Gartnavel Royal Hospital, 1055 Great Western Road, Glasgow, G12 0XH Scotland UK

**Keywords:** Low vision, visual rehabilitation, quality of life, depression, well-being

## Abstract

**Background:**

Visual Rehabilitation Officers help people with a visual impairment maintain their independence. This intervention adopts a flexible, goal-centred approach, which may include training in mobility, use of optical and non-optical aids, and performance of activities of daily living. Although Visual Rehabilitation Officers are an integral part of the low vision service in the United Kingdom, evidence that they are effective is lacking. The purpose of this exploratory trial is to estimate the impact of a Visual Rehabilitation Officer on self-reported visual function, psychosocial and quality-of-life outcomes in individuals with low vision.

**Methods/design:**

In this exploratory, assessor-masked, parallel group, randomised controlled trial, participants will be allocated either to receive home visits from a Visual Rehabilitation Officer (*n* = 30) or to a waiting list control group (*n* = 30) in a 1:1 ratio. Adult volunteers with a visual impairment, who have been identified as needing rehabilitation officer input by a social worker, will take part. Those with an urgent need for a Visual Rehabilitation Officer or who have a cognitive impairment will be excluded. The primary outcome measure will be self-reported visual function (48-item Veterans Affairs Low Vision Visual Functioning Questionnaire). Secondary outcome measures will include psychological and quality-of-life metrics: the Patient Health Questionnaire (PHQ-9), the Warwick-Edinburgh Mental Well-being Scale (WEMWBS), the Adjustment to Age-related Visual Loss Scale (AVL-12), the Standardised Health-related Quality of Life Questionnaire (EQ-5D) and the UCLA Loneliness Scale. The interviewer collecting the outcomes will be masked to the group allocations. The analysis will be undertaken on a complete case and intention-to-treat basis. Analysis of covariance (ANCOVA) will be applied to follow-up questionnaire scores, with the baseline score as a covariate.

**Discussion:**

This trial is expected to provide robust effect size estimates of the intervention effect. The data will be used to design a large-scale randomised controlled trial to evaluate fully the Visual Rehabilitation Officer intervention. A rigorous evaluation of Rehabilitation Officer input is vital to direct a future low vision rehabilitation strategy and to help direct government resources.

**Trial registration:**

The trial was registered with (ISRCTN44807874) on 9 March 2015.

## Background

Low vision is associated with depression [1, 2], reduced quality of life [2, 3] and reduced mobility [2, 4, 5]. Low vision rehabilitation is defined as an amelioration of the lives of individuals with sight loss by improving functional ability and other general aspects, for example, quality of life and psychosocial status [6]. It represents a range of services that operate at the interface of health and social care services to minimise disability and promote independent living. In the United Kingdom, health-based services attempt to help those with low vision make the best use of their remaining eyesight by providing optical and non-optical low vision aids, advice on lighting and contrast enhancement, and onward referral, where appropriate. Social care services tend to take a more holistic approach based on a comprehensive needs assessment, often in the person’s own home. This can result in modifications to the home environment, provision of non-optical aids, mobility training and advice on benefits. Visual Rehabilitation Officers are trained professionals with a 2-year foundation or 3-year degree qualification, who work with people in their homes, often over several sessions, facilitating the learning of new strategies, which include, for example, how to navigate safely, identify objects, read instructions, the use of low vision aids and non-optical aids. A list of further examples is provided in Table 1.

**Table 1:** Summary of support by the Visual Rehabilitation Officer. The table summarises general areas assessed by the Visual Rehabilitation Officer and examples of provision of training and support in each area

Area assessed	Examples of support
Emotional• Emotional state of participant and family • How family members cope with adjustment to the participant’s sight loss.	• Education on eye condition and related emotional, practical and physical difficulties.• Sign-posting to other organisations that can provide emotional support.
Low Vision Function• Functional vision assessment• Best contrast/colour contrast to assist practice in daily living.• Glare sensitivity.	• Eccentric viewing or steady eye strategy training and scanning techniques.• Provision of tinted glasses/peak cap. Training in techniques/aids for watching television.• Training on use of low vision aids, issued by the optometrist• Referral to optometrist.
Lighting• Lighting assessment for areas in and around home	• Provision of specific task lamps, specialist light bulbs.
Personal• Potential difficulties with dressing, grooming and personal hygiene.	• Provision of strategy for identifying and matching colour of clothing, makeup application, hair management, shaving, application of toothpaste on tooth brush, identifying bottles and contents, feminine intimate care.
Medication• Ability to manage medication independently.	• Provision of strategy to administer eye drops, dispense pills from blister packs, systematically search for dropped pill• Provision of dosset boxes, enlarged labels.
Kitchen• Potential difficulties with cooking and meal preparation.	• Training and provision of equipment for cooking. For example, for pouring liquids, knife management and other safety aspects, sequence/timing strategy.
Home• Potential difficulties with household chores, for example, cleaning, tidying, laundry, bed making.	• Training to support tasks such as cleaning, tidying, laundry and bed making. For example, systematic approach for vacuuming and dusting, measuring soap powder/liquid, separating laundry, folding clothing, safe techniques and equipment to avoid burns when ironing, threading needles, changing sheets.
Entitlements• Criteria check for entitlements.	• Assistance with applications. For example, for Disabled Parking badge, bus pass, Disabled rail card, talking magazines/newspapers, British Wireless for the Blind fund, Personal tax allowance, TV licence, accessing utility discount scheme.• Referrals for benefits assessments.
Referrals• Possible need for referral to specialist service.	• Referral to appropriate agency. For example, Occupational therapist, Physiotherapy, Age Concern, Cardiff Institute for the Blind, GP, Diabetic Nurse, Opticians for Wales Eye Care Low Vision Scheme, CAB, Action for Blind, RNIB Benefits, Speech Therapists, Support Groups, talking books, Care & Repair, Sense Cymru, Befriending, Talk and Support, refer to Sight Cymru BME advisor, Highway Maintenance, Hearing Team Cardiff Social Services.
Orientation and Mobility• Potential orientation and mobility difficulties.	• Long cane training, symbol cane issuing and sighted guide training, guide cane training, route and orientation training.• Support for planning travel arrangements. For example, timetables, relevant platform/bus stop/departure gate, arrangements for company provision of Travel Assistance, etc.
Communication• Potential difficulties with telephones, appointments, correspondence, finance management, IT.	Support and advice on the following:• Appropriate telephones and related devices.• Telephone banking, telephone/online shopping.• Note taking and maintaining appointments diary (large print, address guides).• Techniques or equipment suitable to needs for reading, general correspondence, telling the time.• Strategy for accessing bank and money management• Computer technology assistance and referral for IT skills training.

There is evidence that impaired functional status in individuals with visual loss is associated with depression [7–9], psychosocial impact [10–12], risk of falls, decreased mobility [13–15] and higher rates of mental and physical health comorbidities. Partial evidence exists of improved health-related quality-of-life outcomes following visual rehabilitation intervention [16, 17] and improvement in vision-related quality-of-life following visual rehabilitation [16, 18–20]. However, a paucity of high-quality evidence exists regarding the effectiveness of low vision rehabilitation services, particularly with regard to social care provision [6].

The need for evidence to support the low vision rehabilitation service is driven by the necessity to inform direction for healthcare resources, as a result of the growing social and economic challenges faced by those with visual impairment [6]. The burden on such resources is growing with the increase in the elderly population and therefore, the increasing number of individuals with low vision. The evidence gained from this study will inform the design of a definitive trial, which will help shape the development of low vision rehabilitation services in the UK.

The primary aim is to examine the effect of a Visual Rehabilitation Officer intervention (ROI) on self-reported visual function. The secondary aims are to determine the effect of the low vision rehabilitation service on depression, well-being, loneliness, adjustment to visual loss and generic health-related quality-of-life. The relation between the numbers of intervention items received on the outcomes will also be assessed.

## Methods/design

**Fig. 1:**
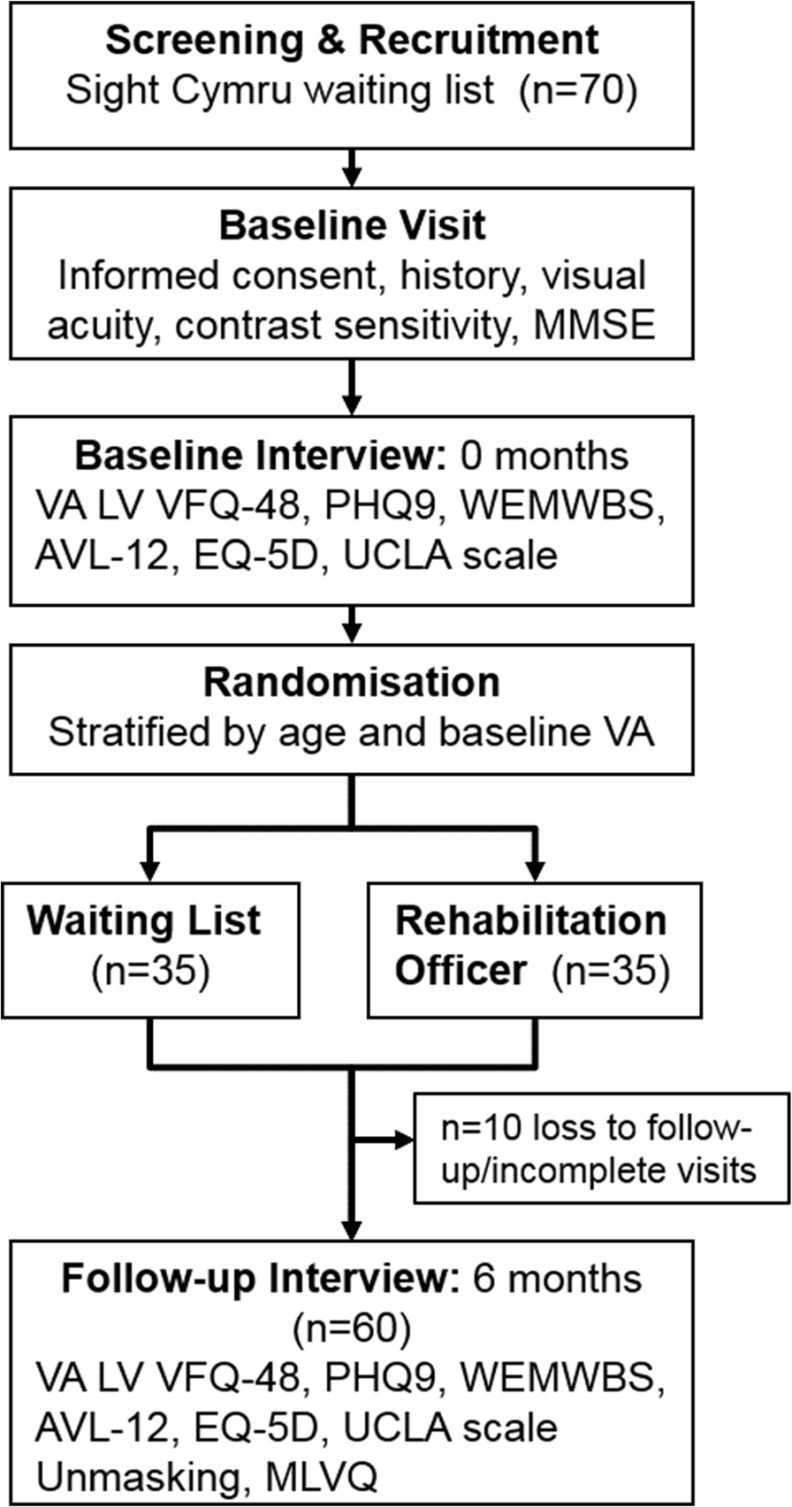
Study flow diagram

This exploratory assessor-masked individually randomised single centre controlled trial is designed to give robust estimates of the effect of a ROI on self-reported functional, psychosocial and quality of life outcome measures. Participants will be allocated to the ROI or waiting list arms of the trial in the ratio 1:1. The trial will be carried out over 18 months (see flow diagram in Fig. 1).

The Research Ethics Audit Committee, at the School of Optometry and Vision Sciences, Cardiff University reviewed and approved the study (#1377).

### Intervention

There is one active intervention in the trial, that is, the ROI. In one to eight sessions, a Visual Rehabilitation Officer will conduct home visits to assess the status of the individual with low vision and provide training and support in specific areas of need.

The support is tailored to the individual in a number of areas including mobility, use of low vision aids, household tasks, communication and administrative tasks. Table 1 details the types of intervention that may be provided by the Visual Rehabilitation Officer. Not all components are provided to each person, because the ROI is tailored to meet individual requirements (Table 1). The aim of the rehabilitation is to promote independence by helping individuals learn new skills or regain lost skills and rebuild confidence following sight loss. This support may be implemented by the provision of information, equipment, encouragement, training and/or referral to other agencies. This type of intervention is typical of that provided by Visual Rehabilitation Officers in the UK.

For each participant in the intervention arm, a list of intervention items implemented and the number of visits will be recorded, by the Visual Rehabilitation Officer (Table 2). In this trial, participants will be recruited from South East Wales. The intervention will be provided by a team of two experienced Visual Rehabilitation Officers (each with 7 to 8 years of experience) based at the Sight Cymru rehabilitation service. This service was chosen for its proximity to the research centre at Cardiff University and the willingness of the service to support a rigorous research study.

**Table 2:** List of intervention items recorded by the Visual Rehabilitation Officer

Intervention items:
Initial assessment
Functional vision assessment
Low vision training/advice
Magnification advice
Lighting advice
Personal care/medication advice
Washing and dressing
Medication management
Kitchen skills
Safe pouring technique
Food preparation/cooking
Cleaning/managing the home
Laundry
Ironing
Money issues
Orientation and mobility training
Road safety check
Sighted guide technique
Symbol cane advice
Guide cane training
Long cane training
Communications
Reading and writing
Accessing TV and radio
Using phones
Telling the time
Audio books
IT
Social and leisure activities
Emotional well-being
Referrals to other agencies (specified)
Other (specified)

The second arm of the study is a ‘waiting list control’ group, consisting of individuals on the existing Sight Cymru waiting list for the visual rehabilitation service. The ‘waiting list control’ provides individuals electing to take part in the study with a better level of care than ‘treatment as usual’ because the service will be received on completion of the study, that is, after 6 months, which is sooner than for those on the existing waiting list.

### Outcome measures

Outcome measures will consist of the questionnaires administered at baseline and 6 months (±1 month) follow-up. The primary outcome measure will be the 48-item Veterans Affairs Low Vision Visual Functioning Questionnaire (VA LV VFQ-48), a validated unidimensional functional outcome measure questionnaire that assesses the difficulty in performing daily activities in visually impaired individuals [21]. Secondary outcome measures will include: the Patient Health Questionnaire (PHQ-9), an assessment of depression symptom severity on a nine-item scale, based on criteria for depressive episodes including concentration problems and suicide [22]; the Warwick-Edinburgh Mental Well-being Scale (WEMWBS), a population measure of subjective well-being, involving 14 positively worded questions about aspects of positive thoughts and feelings [23]; the Adjustment to Age-related Visual Loss Scale (AVL-12), a measure of psychological adjustment to vision loss [24]; the standardised Health-related Quality of Life Questionnaire (EQ-5D), a ‘utility’ measure to assess five health-related dimensions, that is, mobility, self-care, usual activities, pain/discomfort and anxiety/depression [25]; and the UCLA Loneliness Scale, a 20-item scale to measure subjective feelings of loneliness and social isolation [26].

These instruments were selected on the basis of the following evidence. The VA LV VFQ-48 has been assessed as a high-quality instrument for use in evaluating adults with low vision [27]. The PHQ-9 is a suitable measure of depressive symptoms in those with visual loss [28]. Mental well-being is a prominent issue in governmental health policy, and the utility of the WEMWBS has been demonstrated in general populations [29]. The AVL-12 has been adopted effectively in several trials of individuals with low vision following rehabilitation [30–32]. Whilst the EQ-5D may be unresponsive to low vision rehabilitation [33], it is the preferred measure of health utility by the National Institute of Clinical Excellence guidance (NICE article PMG9, 2013). It is acknowledged that health disorders are risk factors for loneliness [34], and the UCLA scale has been used to evaluate individuals with low vision [35].

Outcome measures will be obtained over the telephone by a trained interviewer who will be masked to the group allocation. The order of outcome measures will be partially randomised with a pre-determined order, such that the PHQ-9 and UCLA scale (negatively worded) should be performed before the WEMWBS (positively worded), in order to position a positively worded questionnaire at the end of the interview. However, it must be acknowledged that this may affect the quality of responses to the WEMWBS. Whilst participants will be encouraged not to reveal any information about their group allocation during telephone interviews, any masking violations will be recorded. The success of masking will be assessed, by requiring the interviewer to guess the allocation prior to the start of the final 6-month follow-up. All responses will be coded and double entered.

### Sample size

On the basis of a conservative effect size determined by the VA LV VFQ-48 item scores in a previous study [36], a sample of 30 participants in each group at follow-up can be expected to detect a standardised difference of 0.84 logits between those in the intervention and control groups, with 95 % power and an alpha level of 0.05 (two-tailed). To allow for individuals who may withdraw from the study (approximately 15 %), a total of 70 individuals will be recruited.

### Recruitment

Recruitment will take place over a period of 12 months. Individuals will be identified as potential participants on a consecutive basis from the waiting list of Sight Cymru, having previously been referred to this service. Those who are interested in the study will be screened for eligibility. If eligible, potential recruits will be sent a written information sheet.

It is expected that up to 15 % of participants may be lost to follow-up. In order to minimise attrition, participants will be carefully informed at recruitment of the study requirements, the importance of completion of the study and the value of their contribution.

### Inclusion criteria

Individuals over the age of 18 years who require Visual Rehabilitation Officer input will be included. The Sight Cymru criteria for service provision includes anyone with sight loss that cannot be corrected by glasses and that causes them significant difficulties in carrying out daily tasks, regardless of blind or partial sight registration status.

### Exclusion criteria

To maximise the generalisability of the results, the exclusion criteria will be kept to a minimum. Exclusion criteria include ineligibility for the ROI (those aged < 18 years or living outside of geographical catchment area) or those who have significant need, for example significant risk of injury at home (it would be unethical to randomise those at risk of injury to a 6-month waiting list). These individuals will be moved to a fast-track service. Previous recipients of a comprehensive visual rehabilitation service, since their most recent significant decrease in vision, will not be included. Participants who screen positive for significant cognitive or memory problems according to a shortened version of the Mini-Mental State Exam (MMSE) will be excluded. Participants will not be included if they are unable to use a telephone (for example, caused by very poor hearing, unable to understand English, unable to take part in a 6-month study, and unable to provide informed consent). Individuals with planned cataract extraction over the next 6 months will also be excluded.

### Study procedures: consent, baseline assessments, randomisation and interviews

After receiving the study information sheet, participants will be contacted by the interviewer and invited to attend a baseline visit at the School of Optometry and Vision Sciences. At the baseline visit, further screening for the study criteria will take place. Informed consent will then be collected, in addition to a medical history. Visual acuities (EDTRS) and contrast sensitivity (Pelli-Robson) will be measured. Confidentiality of personal information will be maintained by the interviewer, who will anonymise all data.

The baseline telephone interview will take place within one week of the baseline visit. At the baseline telephone interview, the masked interviewer will explain that the interview results are confidential, prior to performing the baseline questionnaires. Within 1 week of the baseline interview, the participants will be randomly assigned (computer-generated schedule) by site staff, to receive the Visual Rehabilitation Officer intervention or to remain on the Sight Cymru waiting list (control) in the ratio 1:1. In the randomisation process, participants will be stratified by age (older than or younger than 65 years) and baseline visual acuity (better or worse than 1.0 LogMAR). In order to minimise the risk of predicting the group allocations, randomisation will be performed in permuted blocks of two, with random variation of the blocking number. Those assigned to the intervention group will receive their first appointment with the Visual Rehabilitation Officer within 3 weeks (±1 week) of randomisation. All data collection will be conducted by one interviewer, masked to the group allocations. Participants will be reminded not to reveal their group allocation to the interviewer during any mutual communications. All participants will have access to hospital- and community-based low vision optometric assessments; that is, the control and intervention groups will differ only in the receipt of the Visual Rehabilitation Officer visits to the intervention group.

Six months after the baseline telephone interview, a follow-up telephone interview will be conducted by the interviewer. In addition to the follow-up questionnaires, information about any change in circumstance over the past 6 months will be collected, such as a change in eye condition status, change in life events or living situation, change in general health or treatments, or referral to other community support services. After the questionnaire outcomes have been collected, the interviewer will be unmasked by opening a sealed envelope, containing the group allocation for each participant. The interviewer will then assess intervention satisfaction, using items from the Manchester Low Vision Questionnaire [37], a validated questionnaire, consisting of a question about how helpful participants perceive the intervention to be, with five response categories. In addition, the interviewer will pose two open questions asking about aspects of the service with which the participants were satisfied or dissatisfied.

### Analysis

All data will be entered into the Statistical Package for the Social Sciences Ver. 20.0.0.2 (SPSS Inc., Chicago, IL, USA) and recoded such that all items have a consistent valance (that is, all items are valanced positively). Any missing data will be recorded, along with any protocol violations.

The VA LV VFQ-48 baseline data will be analysed with Rasch analysis according to the Andrich Rating Scale model using Winsteps ver. 3.75.0 [38]. Rasch analysis is a probabilistic logistic model, which produces Logit values describing item difficulty and person ability, providing questionnaire scores which are on a true interval scale. A scoring key will be produced allowing conversion of the raw questionnaire scores into an interval level score. Rasch analysis will also be used to confirm instrument unidimensionality and assess reliability of item measures.

Of the secondary outcome measures, the WEMWBS and AVL-12 will be Rasch analysed using the same method as described above in order to obtain item measures, confirm unidimensionality and reliability of items. The remaining secondary outcome measures (PHQ-9, EQ-5D and UCLA scale) will be analysed using standard scoring, for comparable results to previous studies using these measures.

Demographic data will be reported using summary statistics. In order to demonstrate study feasibility, an assessment of participant eligibility, recruitment, retention to follow-up, missing data, adherence to the intervention and acceptability of the intervention will be reported by descriptive data. Successful adherence to the intervention will be defined as completed visits by the Visual Rehabilitation Officer.

Baseline characteristics will be summarised by descriptive statistics. Questionnaire scores at follow-up will be analysed using analysis of covariance (ANCOVA), controlling for the baseline score and the key variables (age, co-morbidities and visual acuity) as covariates. A logistic regression analysis will investigate whether the number of intervention items is associated with the outcomes. Standard diagnostics will be used to check model fit (for example, residual versus fitted plots), and standard transformations (square root, log and square) explored where necessary. The analysis will be complete case and intention to treat. This exploratory study is not powered to identify statistically significant differences; therefore, the results will be interpreted using descriptive statistics and 95 % confidence intervals.

## Discussion

In the United Kingdom, a lack of a unified approach exists in the provision of low vision rehabilitation services, with local differences in the type of providers, specialist skills of the rehabilitation workers, caseloads and waiting times [39]. In addition, variability exists in the classification of low vision rehabilitation in the context of health care and social care.

Given the paucity of high-quality evidence, such as results from randomised controlled trials that demonstrate the effectiveness of low vision rehabilitation services [6], it is problematic for the social sector to support and expand this service. Furthermore, a shortage exists of Visual Rehabilitation Officers, with an estimated 550 practising professionals in the UK [40]. This number is declining, in part, as a result of funding cuts, the lack of provision of training and absence of evidence supporting effectiveness.

Career-specific training for Visual Rehabilitation Officers consists of a foundation or honours degree course. The Universities and Colleges Admissions Service in the UK (UCAS) currently lists such a course at only one UK institution [41] for the academic year 2016/17, and other institutions appear to have discontinued the course.

Whilst several previous studies of low vision rehabilitation have used rigorous study methodology, implemented by randomised controlled trial design [2, 17, 36, 42–46], there are several differences from our protocol. A notable lack of effect of low vision rehabilitation was found in a three-arm randomised controlled trial, consisting of standard clinic-based low vision rehabilitation in a hospital-based, low vision clinic; enhanced rehabilitation with additional home visits; and standard rehabilitation with non-rehabilitation home visits from a 'community care worker' [43]. However, the intervention comprised an ‘add-on’ of rehabilitation home visits in addition to the usual care of the clinic, rather than a multi-disciplinary approach. In contrast, the LOVIT study [36, 44] evaluated outpatient rehabilitation for white male service veterans in the United States and demonstrated effective rehabilitation in individuals with macular disease. Another randomised controlled trial compared usual care from a third sector provider with usual care in addition to vision self-management group therapy [17, 42]. After 12 weeks, some improvement in general health and vision-specific outcomes was evidenced on the basis of results of the SF-36 questionnaire [17, 42]. In a trial involving two clinic-based intervention arms and a waiting list control arm, the profile of mood states improved, for both interventions, but a greater effect for self-management was demonstrated [45, 46]. This effect had a larger impact for individuals who were depressed at baseline [45, 46]. Our study protocol bears several differences to the design of these previous randomised controlled trials, with respect to participant groups, the individuals delivering the intervention, the type of rehabilitation and the range of outcome measures.

Given the wider inclusion criteria of our cohort, it is expected that the results of our study will be applicable to the majority of low vision patients, rather than disease-specific. In addition, our study will include a non-intervention control group, and the referral of participants will not be limited to specific referral pathways. Our study will assess the efforts of two experienced Visual Rehabilitation Officers, who are community-based, as opposed to a clinic-based individual. In the study design of Reeves and colleagues [43], the Visual Rehabilitation Officer focussed on low vision aid (LVA) handling, the use of alternative LVAs and other strategies for enhancing vision; whilst in our study, the emphasis is to address the general areas of need. Although the range of outcome measures in our protocol will include vision-related and health-related metrics, we also intend to include measures of depression, well-being and loneliness.

This prospective study will apply rigorous study design to examine a social care intervention. The strengths of the present protocol include the intention to isolate the effect of the Visual Rehabilitation Officers, by examining sustained outcomes over the medium term of 6 months. An advantageous range of outcome measures will include vision-, health- and psychosocial-related metrics. The limited exclusion criteria will ensure the generalisability of the results. The limitations of the protocol are the exploratory nature, the lack of multiple study sites, the modest sample size and the lack of uniformity in the intervention, which will be tailored to the individual. However, such an intervention reflects real-life practice. Another issue is the potential for respondent fatigue due to the length of the questionnaires. To minimize fatigue, participants will be informed in advance of the expected duration of the interview and will be encouraged to take rest breaks between outcome measures.

Given the uncertain future of Visual Rehabilitation Officers, as a profession in the UK, it is important to evaluate the service, using methods that meet the rigorous standards established by medical research, to build on current evidence. This trial is expected to provide robust estimates of the intervention effect, in order to design a large scale randomised controlled trial to evaluate fully the Visual Rehabilitation Officer intervention.

## Trial status

At the time of submission, this trial is in the process of participant recruitment.

## Abbreviations

AVL-12: Adjustment to Age-related Visual Loss Scale
EQ-5D: Standardised Health-related Quality of Life Questionnaire
MMSE: Mini-Mental State Exam
PHQ-9: Patient Health Questionnaire
ROI: Visual Rehabilitation Officer Intervention
VA: Visual Acuity
VFQ-48: 48-item Veterans Affairs Low Vision Visual Functioning Questionnaire
WEMWBS: Warwick-Edinburgh Mental Well-being Scale
